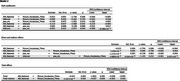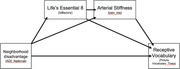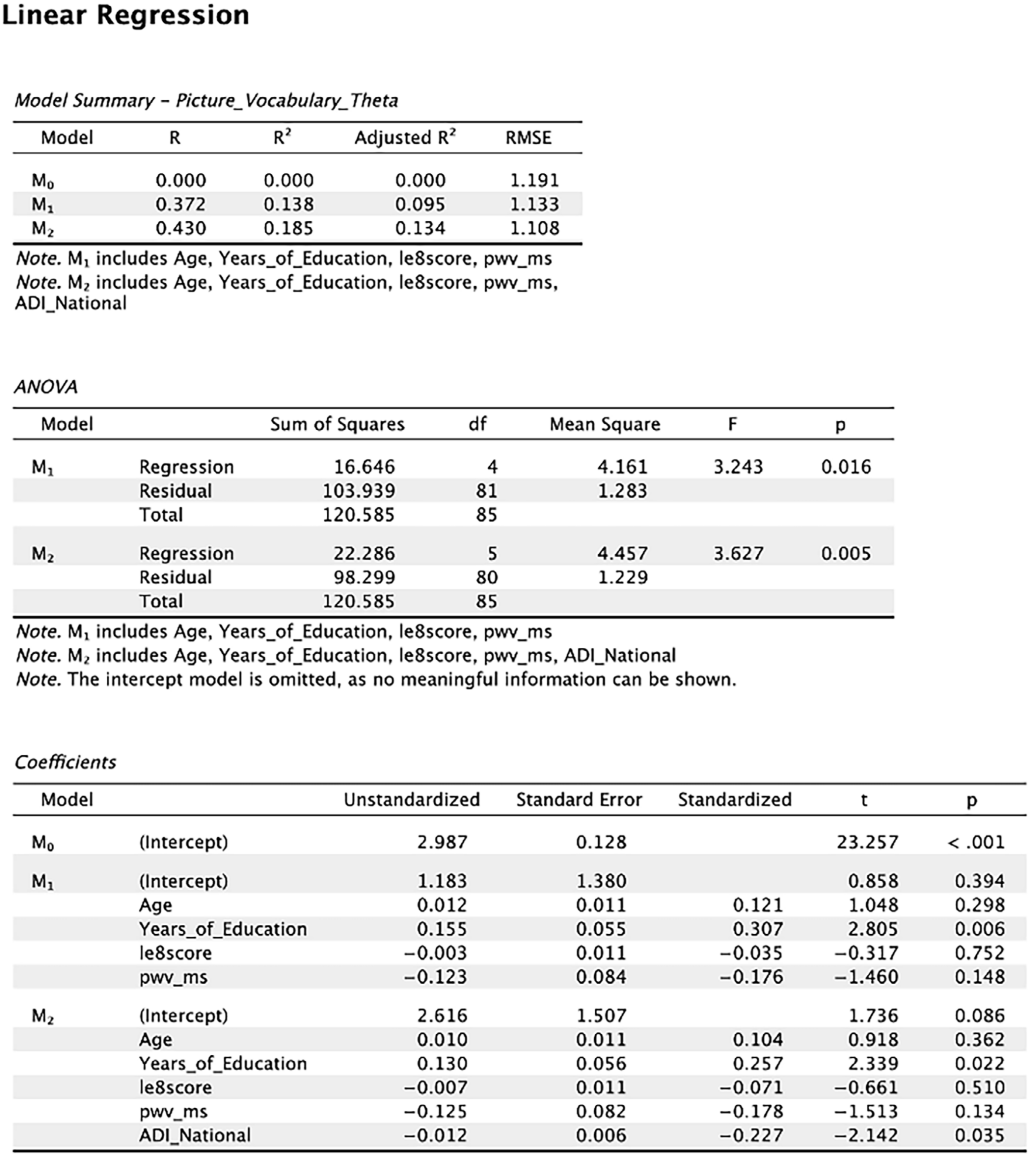# Black Impact 2.0: A 24‐week, community‐based, non‐pharmacological intervention to address cardiometabolic disease and promote brain health in Black Men

**DOI:** 10.1002/alz70860_097393

**Published:** 2025-12-23

**Authors:** Jeremy G Grant, Beniam Daniel Darge, Lauren Hilderbrand, Kaytlin Richardson, Kwame Lartey, Nnanna Ojembe, Amaris Williams, Sir Gregory Powell, Isabella Blum, Stephanie S Ogonuwe, John H Gregory, Timiya S Nolan, Joshua J Joseph

**Affiliations:** ^1^ The Ohio State University, Columbus, OH, USA; ^2^ The Ohio State Wexner Medical Center, Columbus, OH, USA; ^3^ African American Male Wellness Agency, Columbus, OH, USA; ^4^ University of Alabama‐Birmingham, Birmingham, AL, USA

## Abstract

**Background:**

Black Americans are disproportionally affected by dementia and are underrepresented in Alzheimer's disease research. Furthermore, Black men have the shortest life‐expectancy of any race/sex population subgroup in the U.S. and cardiometabolic disease is their leading cause of death. Therefore, there is a pressing need for culturally‐informed interventions to address modifiable risk factors for dementia within this population subgroup. Black Impact is a 24‐week wellness program designed in partnership with academic, community, and government organizations in Central Ohio to promote cardiometabolic health in Black Men.

**Method:**

Black men are screened for cardiometabolic disorders at various community engagement events and enrolled in the program based on their Life's Essential 8 score, a composite measure of blood sugar, hypertension, hyperlipidemia, body mass index, nutrition, smoking, physical activity, and sleep quality. Participants complete 45 minutes of group exercise and 45 minutes of health coaching weekly for 24 weeks. Arterial stiffness is assessed using pulse wave velocity at baseline, week 12, and week 24. Cognitive assessments are performed at baseline and week 24 using selected measures the NIH Toolbox, including receptive vocabulary (Picture Vocabulary), processing speed (Pattern Comparison), cognitive flexibility (Dimensional Change Card Sort), nonverbal reasoning (Visual Reasoning), and memory (RAVLT Immediate and Delayed Recall). Neighborhood disadvantage was assessed using the Area Deprivation Index. Hierarchical linear regression and serial mediation analyses were conducted to examine associations among neighborhood disadvantage, cardiometabolic health, and cognitive performance at baseline.

**Result:**

Participants include 88 Black men (mean = 55.1 years old, 15.4 years of education). Higher neighborhood disadvantage was associated with lower Life's Essential 8 scores and lower receptive vocabulary. Higher arterial stiffness was associated with lower Life's essential 8 scores and poorer cognitive flexibility. Serial mediation analyses showed a significant direct effect of neighborhood disadvantage and receptive vocabulary; the indirect effect via Life's Essential 8 and arterial stiffness was not significant. Hierarchical regression analyses indicated that neighborhood disadvantage predicted receptive vocabulary above and beyond the influence of age, education, Life's essential 8, and arterial stiffness.

**Conclusion:**

Neighborhood disadvantage may provide unique value towards understanding within‐group social determinants of cardiometabolic and cognitive health among Black men.